# Flavor Formation in Chinese Rice Wine (Huangjiu): Impacts of the Flavor-Active Microorganisms, Raw Materials, and Fermentation Technology

**DOI:** 10.3389/fmicb.2020.580247

**Published:** 2020-11-13

**Authors:** Yijin Yang, Wuyao Hu, Yongjun Xia, Zhiyong Mu, Leren Tao, Xin Song, Hui Zhang, Bin Ni, Lianzhong Ai

**Affiliations:** ^1^Shanghai Engineering Research Center of Food Microbiology, School of Medical Instrument and Food Engineering, University of Shanghai for Science and Technology, Shanghai, China; ^2^School of Energy and Power Engineering, University of Shanghai for Science and Technology, Shanghai, China; ^3^Shanghai Jinfeng Wine Co., Ltd., Shanghai, China

**Keywords:** Huangjiu (Chinese rice wine), flavor compounds, microbial community, raw material, fermentation technology, yeast starter, fungi

## Abstract

Huangjiu (Chinese rice wine) has been consumed for centuries in Asian countries and is known for its unique flavor and subtle taste. The flavor compounds of Huangjiu are derived from a wide range of sources, such as raw materials, microbial metabolic activities during fermentation, and chemical reactions that occur during aging. Of these sources, microorganisms have the greatest effect on the flavor quality of Huangjiu. To enrich the microbial diversity, Huangjiu is generally fermented under an open environment, as this increases the complexity of its microbial community and flavor compounds. Thus, understanding the formation of flavor compounds in Huangjiu will be beneficial for producing a superior flavored product. In this paper, a critical review of aspects that may affect the formation of Huangjiu flavor compounds is presented. The selection of appropriate raw materials and the improvement of fermentation technologies to promote the flavor quality of Huangjiu are discussed. In addition, the effects of microbial community composition, metabolic function of predominant microorganisms, and dynamics of microbial community on the flavor quality of Huangjiu are examined. This review thus provides a theoretical basis for manipulating the fermentation process by using selected microorganisms to improve the overall flavor quality of Huangjiu.

## Introduction

Huangjiu (Chinese rice wine), which is brewed with cereal grain, yeast, and *Qu* (a saccharification starter, which is similar to the “*koji*” starter used for making Japanese sake), has a history dating back more than 5,000 years (Varela et al., 2015). Huangjiu has been widely consumed in Asia because of its desirable flavor (Yu et al., 2019). According to the report released by the National Statistics Bureau of China^1^, consumption of alcoholic beverages in China exceeded 800 billion Yuan in 2019, whereas the market share of the Huangjiu industry is significantly smaller than other alcoholic beverages (such as Chinese Baijiu and beer) in China. The lack of flavor diversity and individuation in Huangjiu is responsible for the phenomenon.

**FIGURE 1:**
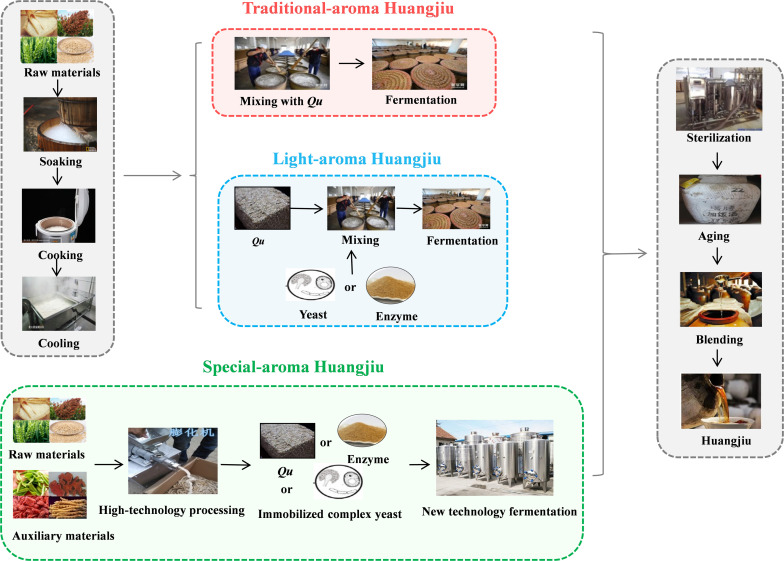
The brewing process of Huangjiu with different aroma types.

As an alcoholic beverage, aroma is the most important factor governing the perceived quality of Huangjiu and consumer preference. Based on aroma types, Huangjiu is divided into traditional-aroma Huangjiu, light-aroma Huangjiu, and special-aroma Huangjiu. Different raw materials and fermentation technologies result in varied aromas of Huangjiu. The production of these three different types of Huangjiu is shown in Figure 1. Generally, Huangjiu is produced by three major processes: selection and pretreatment (soaking) of raw materials, alcoholic fermentation, and post-process treatment. The brewing processes not only affect the fermentation efficiency of Huangjiu, but also largely determine the overall flavor quality of Huangjiu. During soaking, the acid-producing microorganisms use the water-soluble nutrients of rice to grow and produce various acids, which acidify the rice (Adeniran et al., 2012). The acidified rice that contributes to the low pH during initial fermentation can inhibit the growth of miscellaneous bacteria and is conducive to the successful alcoholic fermentation. During the alcoholic fermentation stage, the Huangjiu is firstly fermented at 28°C for 5 days (primary fermentation) and then at 10–15°C for 10–20 days (secondary fermentation; Yang et al., 2019). Primary fermentation assists the growth of yeast, which ferments sugars to ethanol, while secondary fermentation performed under low temperatures increases the accumulation of aroma compounds (Luo et al., 2008; Cao et al., 2010). Finally, the post-process treatment involves sterilization to inactivate microorganisms, thus ensuring the safety and shelf life of Huangjiu, while aging promotes the condensation of acids with alcohols to form esters, which improve the flavor profile of Huangjiu. However, conventional sterilization methods (e.g., boiling at 80–95°C for 15–30 min) lead to large nutritional losses, significant flavor changes, and poor vinosity, all of which are key limitations in the current Huangjiu industry (Yang et al., 2019).

The aroma of Huangjiu is the result of various volatile flavor compounds. Therefore, as the nature, range, and relative concentrations of these compounds change, so do does the aroma and flavor characteristics of Huangjiu. Currently, more than 900 kinds of volatile flavor compounds have been detected in Huangjiu, mainly including esters, alcohols, ketones, aldehydes, phenols, and acids (Chen et al., 2018). As Huangjiu is brewed using a variety of microorganisms co-fermenting under an open environment, the microbial community of Huangjiu during brewing plays a decisive role in the production of flavor compounds (Zhu et al., 2015). Microorganisms detected during Huangjiu brewing are yeast, bacteria, and filamentous fungi; of these, yeast and filamentous fungi contribute the most aroma components due to their involvement in saccharification, liquefaction, and alcoholic fermentation (Huang et al., 2018; Chen C. et al., 2020). Changes in the raw materials or fermentation process directly influence the composition of microbial communities and thereby alter the flavor profile of the resulting Huangjiu. Thus, studying the microbial community composition and changes in the community structure during brewing will improve our understanding of the formation of Huangjiu flavor compounds and lay a theoretical foundation for producing more diverse flavors in Huangjiu.

In this review, we summarize the research on the selection and pretreatment of raw materials, alcoholic fermentation, post-process treatment, and microorganisms that influence the formation of flavor compounds during Huangjiu brewing. We focus on the research that examines the effect of microbial community composition, metabolic function of predominant microorganisms, and changes of microbial community on the flavor quality of Huangjiu. This review will assist brewers in producing Huangjiu with high flavor quality and diversity.

## Selection and Pretreatment of Raw Materials to Improve Huangjiu Flavor

**FIGURE 2:**
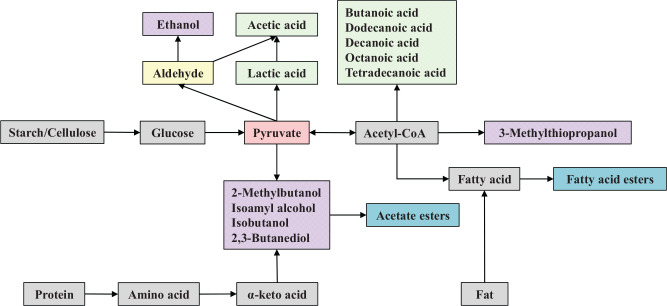
Metabolism of major flavor compounds during Huangjiu brewing.

Raw materials greatly contribute to the flavor by providing microorganisms with precursors of flavor compounds that are crucial for the aroma of Huangjiu (Xu et al., 2018; Chen T. et al., 2020). Rice starch and proteins are correspondingly degraded by microbial enzymes and mostly converted into glucose and amino acids, which effects not only the growth of microorganisms, but also that of the metabolites of microorganisms (Figure 2). Different grains contain different proportions of starch, protein, and fat, so that the resultant Huangjiu exhibits different flavor characteristics. In addition, the pretreatment of raw material (soaking) also has an important effect on the flavor quality of Huangjiu.

### Selection of Grains Appropriate for Huangjiu Fermentation

Grains vary significantly in bitterness, color, and freshness (Shekhawat et al., 2017). Desirable grains used for fermentation need to have a high content of amylopectin, and low contents of protein and fat. The microcrystalline structure of amylopectin in rice is more disordered than that of amylose (Area et al., 2019). Thus, grains containing a high amylopectin content are preferentially used by microorganisms to produce flavor compounds during Huangjiu brewing. Rice varieties also differ in their physicochemical properties, such as the starch granule size, the relative proportion of amylose and amylopectin, and the chain length distribution of polysaccharide (Ahmed et al., 2015). Glutinous rice is considered as the best for producing Huangjiu with a high flavor quality as it contains up to 98% amylopectin (Pachuau et al., 2017). However, due to the high cost and strong taste of the resulting wine, glutinous rice is used for brewing traditional-aroma Huangjiu, such as Hong Qu Huangjiu and some Shaoxing Huangjiu, but is rarely used for brewing light-aroma Huangjiu. Rao et al. (2014) used Yangzhou rice (a type of japonica rice) to ferment Huangjiu and observed that this rice had a higher rate of water absorption than glutinous rice, which resulted in less raw rice remaining after processing. As the use of Yangzhou rice yields Huangjiu with a high concentration of amino acids and a refreshing, lighter flavor, Yangzhou rice is considered a good substitute for glutinous rice in Huangjiu brewing.

The 2-phenylethyl alcohol, which has a unique and pleasant rose-like aroma in Huangjiu, is substantially affected by the raw materials used (Chen et al., 2009; Martínez-Avila et al., 2018). Compared to rice, sorghum and maize produced higher contents of 2-phenylethyl alcohol for Huangjiu brewing. This was attributable to the high content of L-phenylalanine in sorghum and maize, as L-phenylalanine is the substrate for the generation of 2-phenylethyl alcohol and its content in raw materials positively affects the yield of 2-phenylethyl alcohol (Cao et al., 2010; Pineda et al., 2012). When the toxic analog of L-phenylalanine—fluorinated L-phenylalanine is used as a substrate, selecting yeast mutants with resistance to fluorinated L-phenylalanine also allows the increased production of 2-phenylethyl alcohol (Akita et al., 1990; Dueñas-Sánchez et al., 2014; Cordente et al., 2018). In addition, the tannin content of raw materials has been shown to affect the flavor of the resulting Huangjiu. Jia et al. (2018) observed that Huangjiu brewed with sorghum varieties containing different contents of tannin exhibited different flavor characteristics. The differences in fungi community structure induced by different tannin contents may be responsible for the results (Shi et al., 2011).

As consumers demand a diverse range of products, Huangjiu brewing is no longer limited to using commonly consumed grains. The development of new raw materials, such as loquat leaf, oatmeal, barley, and bitter buckwheat, as well as the fungus *Cordyceps militaris*, have become popular topics in Huangjiu brewing research (Yue et al., 2012; Chen and Xu, 2013; Wang et al., 2013; Li H. et al., 2014). Raw materials for Huangjiu brewing are selected not only according to the drinking habits of people in different regions and for economic reasons, but also for enhancing the taste and flavor of Huangjiu. Auxiliary materials can be applied to strengthen the specific flavor characteristics and health benefits of the resultant Huangjiu.

### Effective Soaking Conditions for Rice

Soaking is one of the most important processes during Huangjiu brewing and directly affects the initial fermentation acidity and flavor quality of the resulting wines. The soaking process involves water absorption-mediated expansion of rice, partial decomposition of starch, and acidification of rice (Ji et al., 2013). The total acidity, the concentration of lactic acid, and the lactic acid bacteria (LAB) count in the water after rice soaking are important indicators for evaluating the quality and flavor of Huangjiu.

In traditional brewing of Huangjiu, the rice may be soaked for several weeks, while in modern brewing, soaking is performed for only a few days (Chen et al., 2018; Lv et al., 2018). However, even in modern brewing, a successful soaking process needs to rely on the empirical knowledge of winemakers. The soaking acidity needs to reach a value ranging from 2 g/L to 5 g/L (or soaking 1–3 days in summer and soaking 2–5 days in winter) using modern techniques. Gong et al. (2020) evaluated the effect of different soaking times on rice used for Huangjiu brewing, concluding that the total acidity and amino nitrogen of soaking increased slowly at first, then increased rapidly and finally became stable, while the reducing sugar content exhibited the opposite tendency. Nevertheless, there is still no specific standard regulations for rice soaking, which may result in the inconsistency of quality in terms of taste and flavor among different batches of Huangjiu. In future, standardized soaking procedures should be established to ensure the between-batch flavor stability of Huangjiu.

## Effects of Alcoholic Fermentation on the Flavor of Huangjiu

The principal metabolic process in Huangjiu brewing is the alcoholic fermentation, which consists in the biotransformation of rice nutrients into a wide variety of metabolites responsible for aroma and flavors (Querol et al., 2018). During alcoholic fermentation, fermentation starters of yeast and saccharification starters of *Qu* involved in the principal metabolic process are applied to improve the quality of Huangjiu. However, the starter strains may be sensitive to environmental conditions during the long fermentation period and thus developing high-efficiency fermentation techniques that are suitable with the growth of strains are also necessary.

### Fermentation Starter of Yeast

Within yeast species, *Saccharomyces* strains are the main group that can survive and contribute to wine fermentation. Yeast fermentation not only produces ethanol, but also generates a range of volatile flavor compounds, which result in the specific flavor characteristic of Huangjiu (Fleet, 2003; Chen and Xu, 2012; Cai et al., 2018). As microbial composition during Huangjiu fermentation is complex, a purebred yeast starter is typically used to provide a growth advantage by preventing the excessive proliferation of bacteria, as this can lead to rancidity. However, Yang et al. (2017) found that fermentation performed with inoculation of a single *Saccharomyces* strain leads to a less mellow flavor and taste than that of Huangjiu fermented with two *Saccharomyces* strains. The exception is *Hong Qu* Huangjiu, which is fermented with *Hong Qu* and *Bai Qu* containing a variety of yeasts instead of purebred yeast. But the undefined number of different yeasts makes the brewing of *Hong Qu* Huangjiu uncontrollable. The use of mixed *Saccharomyces* strains has been proven as an effective strategy to improve the flavor profiles and diversity of Huangjiu (Yang et al., 2017). With the knowledge that *non-Saccharomyces* species actively participate during the alcoholic fermentation, the co-fermentation of selected non-*Saccharomyces* with *S. cerevisiae* has been a new strategy to produce beer or grape wine products with more complex aromatic and flavor characters (Escribano-Viana et al., 2018; Zdaniewicz et al., 2020). In this regard, more and more attention should be paid to the use of controlled mixed fermentation with selected *S. cerevisiae* and non-*Saccharomyces* yeasts to change the sensory characteristics of Huangjiu.

**FIGURE 3:**
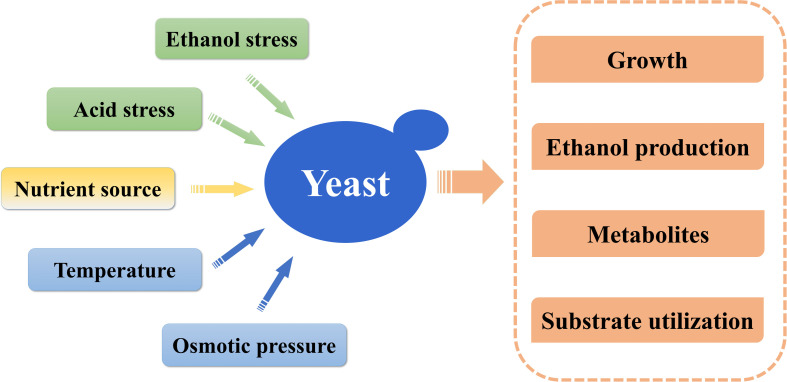
Factors affecting the fermentation of yeasts.

During Huangjiu fermentation, due to nutrients consumption and an accumulation of stressful factors that affect the yeast fermentation (Figure 3), the alcoholic fermentation sometimes remains stuck in the secondary fermentation, thus lowering the quality of the resultant wine (Longo et al., 2020). Among those factors, ethanol stress is the greatest challenge for yeast striving to survive and ferment, as yeast contributes to high ethanol concentration [14–20% (v/v) in the final fermentation mash of Huangjiu], which is also toxic to yeast cells (Chen and Xu, 2012; Snoek et al., 2016). Furthermore, nutrients’ competition among the complex microbial community during secondary fermentation of Huangjiu makes yeast confront higher stress levels. Thus, acquiring yeasts with a high fermentation performance is always desirable to winemakers, as this can theoretically result in more complete fermentation and a higher flavor quality of the resulting wine (Steensels and Verstrepen, 2014). The improvement of fermentation performance and sensory characteristics by yeast has focused on *S. cerevisiae* strains isolated from the natural environment or fermentation foods, along with some directed evolution, mutagenesis, and strain hybridization. Xie et al. (2010) and Yang et al. (2013) screened *S. cerevisiae* strains with a quick fermentation ability and high stress tolerance, which could effectively improve the fermentation efficacy of Huangjiu. *S. cerevisiae* hybrid created by the strategies of directed evolution and protoplast fusion displayed higher flavor production and oenological performance in Huangjiu brewing (Yang et al., 2018). Yeast isolates screened with specific flavor compounds production, such as 2-phenylethanol (De Lima et al., 2018), Isoamyl alcohol ester (Asano et al., 1999), and ethyl caproate (Arikawa et al., 2000; Takahashi et al., 2017), have also been frequently reported in Japanese sake. Screening yeast strains for flavor formation in Huangjiu is a technique that is just now beginning to be used, although it is one that has been widely developed for Japanese sake. The innovative screening strategies of Japanese sake are worth learning from for Huangjiu in the future.

Apart from traditional screening, genetic modification techniques are also capable of creating new yeast strains with specific metabolic profiles for alcoholic fermentation (Krogerus et al., 2017). Ding et al. (2015) enhanced deacidification activity in *Schizosaccharomyces pombe* using genome shuffling, and Dong et al. (2019) increased production of acetate ester in *S. cerevisiae* using a “self-cloning” integration strategy. Ohashi et al. (2020) reported that the removal of feedback inhibition of NAGK activity resulted in the significantly higher production of ornithine in sake and sake cake by gene expression. Although the methods mentioned above can be used in developing new yeasts with novel properties for alcoholic fermentation, genetically modified yeasts are generally not permitted in the production of alcoholic beverages at present. Therefore, only novel strains and innovations discovered by natural breeding will be of immediate use.

### Saccharification Stater of *Qu*

As for the saccharification starter of *Qu*, it could be made either by natural inoculation and artificial inoculation (Ji et al., 2018). Generally, *Qu* is prepared by the spontaneous fermentation of raw materials and inoculated with fungal, yeasts, and bacteria to secrete enzymes such as glucoamylase and protease (Mo et al., 2009). Artificial inoculation of *Qu* is often made by purebred fungus, while natural inoculation of *Qu* contains abundant microorganisms. Based on the color of fungal spores, *Qu* is classified as yellow *Wheat Qu*, red *Hong Qu*, and yellow *Hong Qu*. Aside from *Hong Qu*, in which the dominant fungal species is *Monascus* spp., the other artificial *Qu* are cultivated with *Aspergillus* spp. Of these, the commonly used *Qu* is raw *Wheat Qu* (RWQ), which is made by natural inoculation, and cooked *Wheat Qu* (CWQ), which is made by artificial inoculation. To make RWQ, wheat is squashed and squeezed into bricks, followed by piling up in the natural environment for at least 2 months (Ji et al., 2018). Although RWQ contains abundant microorganisms that may be conducive to generating more flavor compounds, it could not guarantee effective microbial composition and quantity, rendering control of the brewing process difficult and the quality of RWQ inconsistent. CWQ, which is inoculated only with *Aspergillus flavus*, can improve the saccharification rate as well as the fermentation efficiency, and thus has been widely utilized for industrial Huangjiu production. However, purebred CWQ produces weak-flavored Huangjiu, and thus, a combination of CWQ and RWQ is generally applied in the Huangjiu industry. Moreover, to reduce costs and improve efficiency, manufacturers also explore different combinations of *Qu* and enzymes as saccharification starters. Currently, high-temperature α-amylase, medium-temperature α-amylase, composite glucoamylase, and acidic protease are the most commonly used industrial enzymes. The rapid pace of development in enzyme engineering means that an increasing number of enzymes will be available for Huangjiu production.

Microorganisms in *Qu* produce large amounts of cellular metabolic enzymes that subsequently produce small molecules throughout Huangjiu fermentation, which contribute to flavor formation in the final product (Yang et al., 2017). Due to variations in the production process and raw materials for *Qu*, the microorganisms contained therein also vary. Yu et al. (2012) studied the volatile flavor compounds of different traditional Huangjiu and their representative fungus in *Wheat Qu*, and results showed that *Wheat Qu* not only acted as a saccharifying agent, but also increased the activity of yeast and the formation of aroma compounds. In other words, the aroma of Huangjiu can be adjusted according to the amount of *Qu* used in fermentation, implying that differences in the amount of *Qu* could lead to significant differences in the ultimate flavor profile of the wine.

### Different Brewing Techniques for Huangjiu Fermentation

Traditional Huangjiu is successively produced by saccharification and fermentation stages. The fermentation allows starch to be completely hydrolyzed to glucose so that sugar is no longer produced during the fermentation phase, resulting in a decrease in the sugar concentration. However, the high osmotic pressure created by the high initial concentration of glucose inhibits the growth of yeast and thus limits the fermentation rate of Huangjiu (Snoek et al., 2016). Hence, simultaneous saccharification and fermentation, which could improve fermentation efficiency and reduce energy consumption, is applied in the production of modern Huangjiu (Wang et al., 2014). During synchronous fermentation, the sugar produced by amylase-mediated starch hydrolysis is immediately utilized by *Saccharomyces*. This effectively promotes fermentation, and thus increases ethanol yield, which significantly shortens the required entire fermentation time and reduces the possibility of external microbial contamination (Xu et al., 2015). Gong et al. (2013) examined the effects of different brewing processes on physicochemical indicators and the concentration of higher alcohols in Huangjiu. The results showed that the ethanol concentration in Huangjiu fermented by successive steps of saccharification and fermentation was lower than that in Huangjiu from simultaneous fermentation, which was due to the inhibition of yeast activity and a lack of nutrients. In terms of higher alcohols, similar results were also observed between the two fermentation processes. This is because yeast is inhibited by the high concentration of sugar in the pre-fermentation period and thus produces lower content of higher alcohols. That is, during post-fermentation in the two-step process, low concentrations of carbon and nitrogen sources were present, which inhibited yeast activity, resulting in the lack of α-keto acid formation (Takagi, 2019). The resulting large concentrations of ketone acids could not be converted into amino acids, which in turn could not be converted into higher alcohols via the Erlich pathway (Avalos et al., 2013).

Compared with Huangjiu made from conventionally fermented steamed rice with *Qu* and yeast, it has been found that rice liquefied by enzymes and then fermented with *Qu* and yeast exhibited a higher fermentation efficiency (Li et al., 2013). Applying the method of enzyme liquefaction, the pretreatment processes of rice soaking and cooking were both excluded. Bechman et al. (2012) reported that using the liquefaction method to produce Huangjiu could increase the content of higher alcohols and decrease the content of sugar. Furthermore, the content of amino nitrogen was also higher than wine fermented by simultaneous fermentation. This may be attributable to the liquefaction of raw material, which would disperse protein particles throughout the fermented mash, where they are more easily utilized by proteases to increase the amino nitrogen level (Arroyo-Lopez et al., 2009). Although the liquefaction method simplifies the fermentation process and increases the utilization rate of raw materials, the finished wine is less palatable. Hence, the liquefaction method has not been widely applied in the Huangjiu industry.

To prevent environmental pollution of water from soaking, Wei et al. (2017) proposed an innovative brewing technique which involved adding *Lactobacillus* to make up for the total acid originally produced by soaking. Besides, the metabolic reaction of *Lactobacillus* could contribute to the flavor profiles of alcoholic beverages (e. g., malolactic fermentation in grape wine), though the role of *Lactobacillus* in the lavor development of rice wine has not been investigated systematically (Rhee et al., 2011; Oguro et al., 2017). The method could not only save water resources and reduce environmental pollution, but also decrease the content of biogenic amine, which might have deleterious effects on human health if the content is too high (del Rio et al., 2020). Compared to Huangjiu brewed using the rice soaking process, Huangjiu produced with the addition of *Lactobacillus* exhibited a higher content of esters and a lower content of alcohols, which led to a more soft-tasting Huangjiu after a shorter aging time (Regueiro et al., 2017; Wei et al., 2017). Moreover, as there is no standard method for rice soaking, which means that there is inconsistent quality between different batches of Huangjiu, this eco-friendly and simplified brewing technique may be widely applied in the future.

## Post-Process Treatment to Ensure Safety and Enhance Aroma

### Sterilization Techniques

Post-process sterilization is a critical step during Huangjiu production, since it can partially prevent micro-organism contamination, which affects the shelf life and safety of Huangjiu products. Thermal sterilization (boiling at 80–95°C for 15–30 min) is commonly used in the Huangjiu industry. However, this conventional boiling technique not only leads to large nutritional losses, but also significant flavor changes (Xu et al., 2015). Moreover, during boiling, glucose, proteins, polyphenols, and other substances tend to undergo non-enzymatic browning, which affects the flavor and color of the final Huangjiu product (Li X. et al., 2014; Xu et al., 2016). Over the past decades, many studies have been conducted attempting to overcome the limitations of traditional boiling, and a series of non-thermal sterilization technologies have been developed for Huangjiu (Chang, 2003; Yang et al., 2019). Notably, high hydrostatic pressure (HHP) technology inactivates foodborne spoilage and pathogenic microorganisms without causing the significant loss of sensory and nutritional value of food products. Moreover, Tian et al. (2016) concluded that HHP could shorten the aging time of Huangjiu significantly and improve wine quality. In another study reported by Yang et al. (2019), it was found that HHP treatment appeared to be more beneficial for the total amount of aroma-active volatiles in Huangjiu than thermal treatment. Thus, non-thermal sterilization techniques, such as HHP processing, can effectively enhance the flavor profiles of Huangjiu, but the high cost of HHP equipment limits the wide use of this technique in the Huangjiu industry. As wineries are focused on how to improve the flavor quality of their wine products, non-thermal sterilization techniques will become prominent sterilization techniques in the future.

### Formation of Key Flavor Compounds During Aging

As fresh wine has an insufficient aroma and a rough taste, it usually requires a period of storage, commonly known as aging, to reduce its pungency and render it more palatable, full-bodied, and well-balanced in flavor (Tao et al., 2014). Huangjiu is commonly stored in traditional pottery jars for 1 to 3 years during the aging process, with high-quality Huangjiu aged for longer periods (Fan and Xu, 2012). Due to the high energy consumption and uncontrollability of this natural aging process, it has failed to meet the market demand. Consequently, stainless-steel pots are currently used for Huangjiu aging. However, the flavor characteristics of Huangjiu aged in a pottery jar are considerably different from those of Huangjiu aged in a stainless-steel pot at an industrial scale (Huang et al., 2020). Therefore, understanding the key flavor compounds and their formation mechanisms is crucial for optimization of the aging process of Huangjiu. Chen et al. (2019) reported that vanillin, 3-methylbutanol, sotolon, and benzaldehyde contributed substantially to the overall aroma of aged Huangjiu. During aging, the content of aldehydes, ketones, lactones, and phenolic flavor compounds changed consistently with aging time. The concentrations of key aroma compounds such as benzaldehyde, 3-methylbutanol, 1,1-diethoxyethane, sotolon, and vanillin increased significantly with the aging time, while those of other key aroma compounds, such as 4-vinylguaiacol and methional, decreased significantly with aging time. However, the formation mechanisms of these key flavor compounds remain to be elucidated.

Aging involves two processes: physical maturation and chemical maturation (Tao et al., 2014). During physical maturation, the hydrogen bonding among flavor compounds of Huangjiu and the volatilization of heterogeneous volatile compounds may contribute to the final flavor characteristic of Huangjiu (Cao et al., 2018; Wang et al., 2019). As for chemical maturation aging, a variety of chemical reactions, such as oxidation, esterification, and hydrolysis reactions, may affect the composition of flavor compounds, and thus are responsible for the flavor quality of Huangjiu. Generally, spiciness, roughness, and bitterness, which are associated with the “unpleasant” flavor characteristics, are prominent in fresh wine due to its excessive ethanol content (Jones P. R. et al., 2008). After aging, the increased acidity promoted proton exchange between water and ethanol and thus strengthened the hydrogen-bonding structure in alcoholic beverages (Nose et al., 2004, 2005). This reduced the freedom degree of ethanol molecules and produced a softer tasting Huangjiu. Furthermore, alcohol, aldehydes and other substances in wine may be oxidized to acid, which can then react with alcohols to afford esters and thus enhance the aroma of Huangjiu (Shen et al., 2011). Currently, there are many other new techniques that have been proposed for aging, such as microwave aging and biological aging (Roldán et al., 2017; Zhong et al., 2020). Nevertheless, these techniques have different effects on the aroma and taste of the resultant Huangjiu (Zhang et al., 2005), which is also a major challenge for the Huangjiu industry.

## Relationship Between Microbial Community and Huangjiu Flavor

The composition of microbial communities in Huangjiu is complicated. This is due to microorganisms derived from the yeast starter and *Qu*, the open fermentation environment, and the introduction of environmental microorganisms by the re-use of rice soaking water, which increases the diversity and complexity of the microbial communities in Huangjiu (Chen C. et al., 2020). Owing to the interaction of microorganisms in different fermentation stages of Huangjiu, different types and contents of flavor compounds are produced (Figure 2), which greatly affect the flavor characteristic of Huangjiu. Generally, alcohols account for more than 50% of the total flavor compounds content in Huangjiu, with esters being the second most abundant flavor compounds (Chen et al., 2018). Alcohols are mainly generated by yeast metabolism, and although they usually have a low odor activity value, their high concentration means they play a key role in the process (Yang et al., 2018). Esters constitute one of the most important classes of flavor compounds because they are largely responsible for the desired fruity, candy, and perfume-like aroma associated with Huangjiu (Hu et al., 2018). Esters can form from alcohols and acids in the absence of enzymes and microorganisms. However, this manner of ester formation is apparently too slow to account for the large amounts of ester normally found in alcoholic beverages. Thus, the enzymic formation of esters by microorganism metabolism is the main manner through which to accumulate esters for Huangjiu. As most of the flavor compounds are produced by the common metabolism of multiple microorganisms, the compositions of the microbial community directly affect the metabolic function of microorganisms and the production of flavor compounds during Huangjiu brewing.

### Metabolic Function of Dominant Microorganisms During Huangjiu Brewing

Microorganisms that have been detected in Huangjiu are listed in Table 1, with yeast, molds, and bacteria being the main types that have been identified during Huangjiu brewing. Chen and Xu (2012) found that the dominant flavor compounds in Huangjiu, including alcohols, some esters, and volatile acids, were mainly produced by yeast fermentation. During Huangjiu brewing, *S. cerevisiae* is not the only yeast that can contribute to the flavor of wine. *Non-Saccharomyces* yeasts also contribute positively to the brewing process as they produce certain additional aromatic compounds that improve the flavor and aroma of wine (Zot et al., 2008; Jolly et al., 2014). A *Saccharomyces*-yeast used for Huangjiu production should exhibit a strong fermentation ability, reproduce rapidly, generate a high concentration of alcohol with less foaming, and be strongly resist to bacterial contamination. In recent years, *AS.2.1392* (original 501, Shanghai Jinfeng brewery), *M85* (Wuxi brewery), and *zmyl-6* (Zhejiang Institute of Microbiology and Shaoxing brewery) have been demonstrated to show excellent fermentation performances, and have been popular in Huangjiu production. In terms of *non-Saccharomyces* yeast, *Debaryomyces*, *Issatchenkia*, *Meyerozyma*, *Rhodotorula*, *Wickerhamomyces*, *Blastobotrys*, *Candida*, and *Clavispora* are the most commonly identified *non-Saccharomyces yeasts* in Huangjiu (Table 1). When amino acids constitute the major nitrogen source, *non-Saccharomyces* yeasts convert tryptophan and L-phenylalanine into tyrosol, tryptizol, and 2-phenylethyl alcohol via Ehrlich pathway metabolism (Hazelwood et al., 2008; de Jesús Rodríguez-Romero et al., 2020). These alcohols not only affect the flavor and taste of wine, but are also involved in the growth regulation of yeast (Avbelj et al., 2015). Aromatic alcohols could act as quorum-sensing molecules, as they are recognized by other yeast cells when secreted into the extracellular medium, which induces the pseudo-filamentous growth of yeast (Chen and Fink, 2006). Canonico et al. (2019) found that using non-*Saccharomyces* yeast strains in sequential fermentation with *S. cerevisiae* could produce a Chardonnay wine with reduced ethanol concentration and acceptable chemical volatile profiles. Binati et al. (2020) reported that the use of selected non-*Saccharomyces* strains in conjunction with *S. cerevisiae* positively modulated some relevant chemical parameters and improved the aromatic intensity of Pinot Grigio grape wine. Although non-*Saccharomyces* yeasts can provide a means for increasing aroma and flavor diversity in fermented beverages, it is rarely selected and applicated in rice wine brewing, let alone in Huangjiu. Screening novel non-*Saccharomyces* isolates and evaluating their contribution to the sensory characteristics of rice wine will help to differentiate the final products.

**TABLE 1:** Microorganisms detected in Huangjiu during brewing by HTS technology.

Microbes	Genus level	Sample origin	References	Identified methods
Bacterium	*Saccharopolyspora*	SXRW	Xie et al., 2013	Illumina sequencing
	*Streptomyces*	SXRW	Xie et al., 2013	Illumina sequencing
	*Mycobacterium*	SXRW	Xie et al., 2013	Illumina sequencing
	*Arthrobacter*	SXRW	Xie et al., 2013	Illumina sequencing
	*Actinosynnema*	SXRW	Xie et al., 2013	Illumina sequencing
	*Amycolatopsis*	SXRW	Xie et al., 2013	Illumina sequencing
	*Kocuria*	SXRW	Xie et al., 2013	Illumina sequencing
	*Rhodococcus*	SXRW	Xie et al., 2013	Illumina sequencing
	*Bacillus*	SXRW	Lv et al., 2013; Xie et al., 2013	Illumina sequencing
	*Staphylococcus*	SXRW	Xie et al., 2013	Illumina sequencing
	*Lactobacillus*	SXRW	Lv et al., 2013; Xie et al., 2013	Illumina sequencing
	*Pantoea*	SXRW	Lv et al., 2013; Xie et al., 2013	Illumina sequencing
	*Burkholderia*	WYQRW and GTQRW	Li et al., 2018	16S rRNA genes
	*Erwinia*	WYQRW and GTQRW	Li et al., 2018	16S rRNA genes
	*Klebsiella*	WYQRW and GTQRW	Lv et al., 2013; Li et al., 2018	16S rRNA genes
	*Ochrobactrum*	WYQRW and GTQRW	Li et al., 2018	16S rRNA genes
	*Shewanella*	WYQRW and GTQRW	Li et al., 2018	16S rRNA genes
	*Agrobacterium*	WYQRW and GTQRW	Li et al., 2018	16S rRNA genes
	*Acinetobacter*	WYQRW and GTQRW	Li et al., 2018	16S rRNA genes
	*Lactococcus*	WYQRW and GTQRW	Lv et al., 2013; Li et al., 2018	16S rRNA genes
	*Brevibacillus*	WYQRW	Huang et al., 2018	16S rRNA genes
	*Gluconacetobacter*	WYQRW	Huang et al., 2018	16S rRNA genes
	*Pseudomonas*	WYQRW	Lv et al., 2013; Huang et al., 2018	16S rRNA genes
	*Raoultella*	WYQRW	Huang et al., 2018	16S rRNA genes
	*Serratia*	WYQRW	Huang et al., 2018	16S rRNA genes
	*Sphingomonas*	WYQRW	Huang et al., 2018	16S rRNA genes
	*Staphylococcus*	WYQRW	Huang et al., 2018	16S rRNA genes
	*Thermus*	WYQRW	Huang et al., 2018	16S rRNA genes
	*Weissella*	WYQRW	Lv et al., 2013; Huang et al., 2018	16S rRNA genes
	*Enterobacter*	ZJRW	Fang et al., 2015	Illumina sequencing
	*Acidovorax*	ZJRW	Fang et al., 2015	Illumina sequencing
	*Propionibacterium*	ZJRW	Fang et al., 2015	Illumina sequencing
Fungi	*Monascus purpureus*	WYQRW and GTQRW	Li et al., 2018	ITS1 regions
	*Saccharomyces sp*	WYQRW and GTQRW	Li et al., 2018	ITS1 regions
	*Aspergillus niger*	WYQRW and GTQRW	Li et al., 2018	ITS1 regions
	*Eurotionmycetes sp*	WYQRW and GTQRW	Li et al., 2018	ITS1 regions
	*Fusarium pseudensiforme*	WYQRW and GTQRW	Li et al., 2018	ITS1 regions
	*Rhizopus microsporus*	WYQRW and GTQRW	Li et al., 2018	ITS1 regions
	*Aspergillus sp*	WYQRW and GTQRW	Jia et al., 2018; Li et al., 2018	ITS1 regions
	*Agaricomycetes sp*	WYQRW and GTQRW	Li et al., 2018; Rui et al., 2019	ITS1 regions
	*Cunninghamella*	WYQRW	Huang et al., 2018	ITS1 regions
	*Cladosporium cladosporioides*	ZJRW	Yu et al., 2012	Illumina sequencing
	*Alternaria alternata*	ZJRW	Yu et al., 2012	Illumina sequencing
	*Penicillium sp*	ZJRW	Yu et al., 2012	Illumina sequencing
	*Clavispora lusitaniae*	ZJRW	Yu et al., 2012	Illumina sequencing
	*Issatchenkia orientalis*	ZJRW	Yu et al., 2012	Illumina sequencing
Yeast	*Geotrichum*	WYQRW	Huang et al., 2018	ITS1 regions
	*Debaryomyces*	WYQRW	Huang et al., 2018	ITS1 regions
	*Issatchenkia*	WYQRW	Huang et al., 2018	ITS1 regions
	*Meyerozyma*	WYQRW	Huang et al., 2018	ITS1 regions
	*Rhodotorula*	WYQRW	Huang et al., 2018	ITS1 regions
	*Wickerhamomyces*	WYQRW	Huang et al., 2018	ITS1 regions
	*Blastobotrys*	WYQRW	Huang et al., 2018	ITS1 regions
	*Candida*	WYQRW	Huang et al., 2018	ITS1 regions
	*Clavispora*	WYQRW	Huang et al., 2018	ITS1 regions

*SXRW, Shao Xing Huangjiu; WYQRW, Wuyi *Hong Qu* Huangjiu; GTQRW, Gutian *Hong Qu* Huangjiu; and ZJRW, Zhen Jiang Huangjiu.*

Molds produce many enzymes involved in cellular metabolism and the resultant small molecules contribute to the formation of esters (Cai et al., 2018). The molds in Huangjiu are mainly derived from *Qu*, and the dominance of different molds in different samples of *Qu* results in the unique local flavor of Huangjiu (Mo et al., 2009). Researchers have screened functional saccharification and fermentation mold strains from *Qu* in different regions of China and found that *Rhizopus* and *Aspergillus* play crucial roles. *Rhizopus* produces a variety of enzymes, such as high-activity amylase and saccharification enzymes, which play an important role in the saccharification process of Huangjiu (Lücke et al., 2019). At the same time, some *Rhizopus* spp. can process alcohols under certain conditions and thus produce flavor compounds, such as 2-phenethyl alcohol, and the esters ethyl caproate, and ethyl lactate (Londoño-Hernández et al., 2017). *Aspergillu*s can produce acidic proteases and carboxypeptidases during Huangjiu brewing. These proteases hydrolyze the proteins in rice into nitrogen sources, such as peptides and amino acids, that can be easily used by yeast (Chen T. et al., 2020). They can be used as nutrients for yeast growth or as precursors for the synthesis of flavor compounds (Chang et al., 2015). Generally, *Aspergillus* is the most abundant filamentous fungus and is present in various fermentation stages of *Wheat Qu* Huangjiu (Rui et al., 2019). Ji et al. (2018) reported that filamentous molds showed significant differences in Huangjiu fermented with different *Wheat Qu*. Fungal community structure and diversity are affected by organic acids, suggesting that the metabolites of filamentous molds may contribute significantly to the formation of major flavor compounds in Huangjiu. Furthermore, Huangjiu fermentation often increases the contents of ethanol and organic acid due to the lack of sufficient oxygen, which may ultimately lead to the death of bacteria or the loss of the metabolic activity of *Aspergillus*.

Compared with yeast and mold, few studies about the function of bacteria in Huangjiu have been reported. However, the number of different types of bacteria in the microbial community of Huangjiu far exceed the number of types of yeast and mold. Both the traditional PCR-DGGE method and high-throughput sequencing (HTS) analysis have shown that *Bacillus sp.* and LAB are the main bacterial genera present during Huangjiu brewing (Lv et al., 2013; Cai et al., 2018; Huang et al., 2018). A significant change in the bacterial community occurs during brewing, especially in the abundance of *Bacillus* and *Lactobacillus* species. *Bacillus* secrete various hydrolases, which may generate nitrogen-containing flavor compounds, such as pyrazines (Bednarek et al., 2019). *Bacillus* spp. survive as spores in unfavorable environments, which helps these organisms to produce flavor compounds under high ethanol conditions during the secondary fermentation of Huangjiu. LAB produce various antibacterial substances that inhibit pathogens and toxin-producing spoilage organisms (Castellano et al., 2008; Jones R. J. et al., 2008). Organic acids produced by LAB provide precursor substances for the generation of flavor compounds. Wang et al. (2014) reported that the presence of LAB was positively related to the presence of organic acids during Huangjiu brewing.

### Dynamics of Microbial Community During Huangjiu Brewing

The brewing process of Huangjiu is comprised of two stages: primary fermentation at 28°C for 5 days and secondary fermentation at 15°C for 10–20 days under an open environment. The microbial community interacts with the open fermentation environment, and thus changes constantly during Huangjiu brewing. Both fungal and bacterial communities varied significantly in different fermentation periods of Huangjiu (Huang et al., 2018). Numerous studies on the microbial communities of *Wheat Qu* Huangjiu and *Hong Qu* Huangjiu have been reported, as well as on *Qu* and other traditional starters (Wang et al., 2014; Hong et al., 2016; Huang et al., 2018; Ji et al., 2018; Shuang et al., 2019; Chen C. et al., 2020).

At the initial fermentation, owing to the sufficiency of nutrients and a favorable environment, fungal microorganisms grow rapidly to generate a complex fungal community (Yasuda et al., 2012). With the release of some compounds that are harmful to the growth of microorganisms and the consumption of nutrients, the diversity of the fungal community gradually decreases (Kobayashi et al., 2014). During the final stage of fermentation, the populations of some fungal microorganisms increase, which is largely ascribable to the utilization of residual nutrients released during the autolysis of dead cells. Although the diversity of fungal communities changes during the fermentation period, the changes in the dominant genera and their relative abundance are not significant. *Saccharomyces*, *Saccharomycopsis*, *Rhizopu*s, *Monascus*, *Pichia*, *Wickerhamomyces*, *Candida*, and *Aspergillus* have been found to be the predominant genera during the traditional fermentation process of *Hong Qu* Huangjiu, while *Aspergillus*, *Thermomyces*, and *Rhizopu*s play a dominant role in the brewing of *Wheat Qu* Huangjiu (Huang et al., 2018; Liu et al., 2019). Ji et al. (2018) found that *Aspergillu*s species were more abundant in *Wheat Qu* than in various fermentation mashes, and that their abundance continually decreased until the end of *Wheat Qu* Huangjiu fermentation. Normally, the major flavor compounds in Huangjiu are generated by the metabolism of fungal microorganisms (Chen and Xu, 2012; Xie et al., 2012). Mu et al. (2016) found that the production of ethanol and higher alcohols was due to the metabolism of yeast. Furthermore, higher alcohols and organic acids reacted to form esters, and alcohols were oxidized to aldehydes, resulting in an increase of ester and aldehyde contents. During secondary fermentation, the ethanol-producing ability of yeast is reduced by the presence of high concentrations of ethanol and low pH, so that the production of alcohols, esters, and aldehydes is also decreased.

The bacterial communities in Huangjiu has previously been overlooked, however, these organisms not only affect the fermentation efficiency, but also play a decisive role in the flavor quality of Huangjiu. The diversity of bacterial community increases at the initial fermentation and then gradually becomes stable, and the changing environmental conditions may be responsible (Wang et al., 2014). The presence of sufficient nutrients and oxygen and a low content of ethanol are all conducive to the growth of microorganisms during primary fermentation. Conversely, the low temperature and high content of ethanol during secondary fermentation inhibits the growth of microorganisms. The diversity of the bacterial community is much greater than that of the fungal community during Huangjiu brewing (Huang et al., 2018; Chen C. et al., 2020). In the early stages of this research area, studies on the fermented mash of Shaoxing Huangjiu were conducted based on traditional isolation methods, but researchers obtained different results. Zhang et al. (2013) found that *Lactobacillus brevis* occurred during the entire brewing process of Huangjiu, while Hu et al. (2009) identified that *Bacillus subtilis* appeared throughout the whole fermentation process. The limitations of traditional isolation methods mean that they cannot fully reflect the composition of bacterial communities in Huangjiu brewing. Using PCR-DGGE and HTS technologies, researchers confirmed that a number of uncultured bacteria exist in the fermentation broth of Huangjiu, and also delineated their functional metabolism characteristics (Lv et al., 2013; Huang et al., 2019; Shuang et al., 2019). At the genus level, *Bacillus*, *Saccharopolyspora*, *Staphylococcus*, *Lactobacillus*, *Leuconostoc*, *Lactococcus*, *Weissella*, *Pseudomonas*, *Thermoactinomyces*, and *Enterobacteria* were the most abundant bacteria in Shaoxing Huangjiu and Shanghai Huangjiu (both were *Wheat Qu* Huangjiu, but used different types of *Qu*; Ji et al., 2018). As for *Hong Qu* Huangjiu, Huang et al. (2018) reported that *Lactobacillus*, *Bacillus*, *Leuconostoc*, *Lactococcus*, *Raoultella*, *Staphylococcus*, *Pediococcus*, and *Weissella* were the predominant genera. Although there are many different types of *Qu*, LAB play a crucial role in flavor generation of all Huangjiu. The bacterial community is dominated by the genus of *Bacillus*, *Staphylococcus*, and *Thermoactinomyces* during the primary fermentation of Huangjiu, but these are replaced by LAB during secondary fermentation, due to the facultative anaerobic and acid-tolerant features of most LAB (Hong et al., 2016; Shuang et al., 2019; Chen C. et al., 2020). The organic acids produced by LAB increase the acidity and lower the pH value, which inhibits the excessive growth of miscellaneous bacteria to prevent the rancidity of Huangjiu. The autolysis of bacteria also produces peptides, a small amount of amino acids, and other ingredients, which contribute to the aroma and taste of Huangjiu. Based on metagenomics and multivariate statistical analysis, Chen C. et al. (2020) found that *Pediococcus* and *Weissella* showed a strong correlation with the acid-producing ability of microbial communities in Shaoxing rice wine. Huang et al. (2018) found that the generation of fruit-flavored esters were strongly associated with *L. brevi*s and *L. alimentarius*, as well as *Bacillus* and *Lactobacillus*, which contributed substantially to the formation of fatty acid ethyl esters.

## Summary and Perspectives

Huangjiu is a national drink in China, and is thus the most promising wine for exportation. However, the consumer acceptance and market share of Huangjiu are significantly lower than that of Baijiu and beer due to its limited flavor diversity and individuation. As the fermentation of Huangjiu involves multiple microorganisms under an open environment, manipulation of the fermentation process become difficult. Thus, understanding the nature of the microbial communities involved in different fermentation processes is of great significance to improve the flavor quality and economic competitiveness of Huangjiu products. In recent decades, the raw materials, brewing process, and microbial communities of Huangjiu have been comprehensively investigated. The characteristic flavor compounds, the predominant micro-organisms, and their metabolic functions during Huangjiu brewing are now clearer than before. Moreover, many functional strains have been isolated and microbes that were considered uncultivable in the past have now been isolated and cultured.

The contribution of microorganisms to flavor compound mixtures are studied mainly by HTS and multivariate statistics analysis. However, the relationship between microorganisms and flavor compounds based on correlation analysis cannot reveal the function of specific microbes in the fermentation process of Huangjiu. Furthermore, the interaction between microorganisms during Huangjiu brewing also remains to be elucidated. In future, with the further development of HTS technology and the combined application of multi-omics technologies (metagenomics, proteomics, flavoromics, and metabolomics), the aroma-producing microorganisms of microbial communities during Huangjiu brewing may be identified and applicated in the production of high-quality, flavorful Huangjiu. In addition, the investigation of interactions between aroma-generating microorganisms and their adaption to changes in the fermentation environment will provide a more theoretical basis for manipulating the fermentation process, such as by using selected microorganisms to improve the overall flavor quality of Huangjiu.

## Author Contributions

YY: investigation, software, visualization, and writing – original draft. WH: resources, methodology, and visualization. YX: conceptualization, project administration, and writing – review and editing. ZM: resources, visualization, and writing – review and editing. LT: visualization and investigation. XS: writing – review and editing and visualization. HZ: writing – review and editing. BN: writing – review and editing. LA: supervision, writing – original draft, and writing – review and editing. All authors contributed to the article and approved the submitted version.

## Conflict of Interest

HZ and BN were employed by the company of Shanghai Jinfeng Wine Co., Ltd. The remaining authors declare that the research was conducted in the absence of any commercial or financial relationships that could be construed as a potential conflict of interest.

## Footnotes

1 http://lwzb.stats.gov.cn/pub/lwzb/gzdt
