# Corrigendum: Flavor Formation in Chinese Rice Wine (Huangjiu): Impacts of the Flavor-Active Microorganisms, Raw Materials, and Fermentation Technology

**DOI:** 10.3389/fmicb.2021.636810

**Published:** 2021-02-25

**Authors:** Yijin Yang, Wuyao Hu, Yongjun Xia, Zhiyong Mu, Leren Tao, Xin Song, Hui Zhang, Bin Ni, Lianzhong Ai

**Affiliations:** ^1^Shanghai Engineering Research Center of Food Microbiology, School of Medical Instrument and Food Engineering, University of Shanghai for Science and Technology, Shanghai, China; ^2^School of Energy and Power Engineering, University of Shanghai for Science and Technology, Shanghai, China; ^3^Shanghai Jinfeng Wine Co., Ltd., Shanghai, China

**Keywords:** Huangjiu (Chinese rice wine), flavor compounds, microbial community, raw material, fermentation technology, yeast starter, fungi

In the original article, there was a mistake in Figure 2 as published. “2-Methy-butanol” has been corrected as “2-Methylbutanol,” and “Isoamylol” has been corrected as “Isoamyl alcohol.” The corrected Figure 2 appears below.

**Figure 2:**
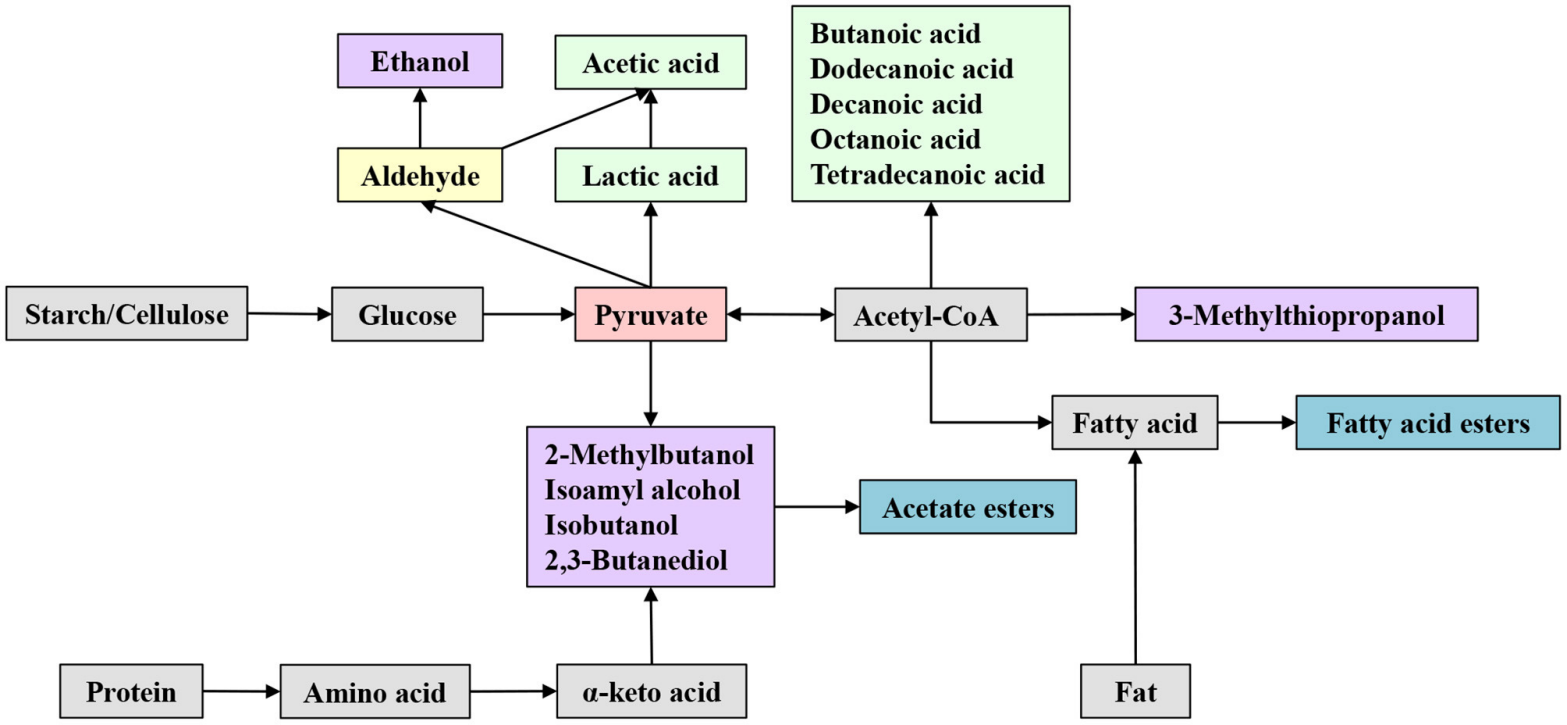
Metabolism of major flavor compounds during Huangjiu brewing.

The authors apologize for this error and state that this does not change the scientific conclusions of the article in any way. The original article has been updated.

